# Clinical Value of Circulating Endometrial Cells in the Diagnosis and Stratified Diagnosis of Endometriosis

**DOI:** 10.3390/jcm15083021

**Published:** 2026-04-15

**Authors:** Shang Wang, Buyun Li, Xue Ye, Qianchen Tai, Hongyan Cheng, Honglan Zhu, Huiping Liu, Xiaoting Wei, Jingjing Gong, Xiaohua Zhou, Xiaohong Chang

**Affiliations:** 1 Department of Obstetrics and Gynecology, Peking University People’s Hospital, Beijing 100044, China; wangshang0515@163.com (S.W.); 2311210287@stu.pku.edu.cn (B.L.); yexuemail@sina.com (X.Y.); chyan7581@sina.com (H.C.); honglanzhu01@163.com (H.Z.); yxylhp@pku.edu.cn (H.L.); wzlwxt@163.com (X.W.); gongjingjing@pkuph.edu.cn (J.G.); 2 School of Mathematical Sciences, Peking University, Beijing 100871, China; pkutqc@pku.edu.cn; 3 Department of Biostatistics, Peking University, Beijing 100871, China

**Keywords:** endometriosis, circulating endometrial cell (CEC), liquid biopsy, imperfect reference standard, stratified diagnosis

## Abstract

**Background/Objectives**: The diagnosis of endometriosis (EM) remains challenging due to the lack of a perfect diagnostic standard and the poor concordance between clinical symptoms and lesion severity. Although laparoscopy is widely used in clinical practice, it is invasive and associated with a non-negligible false-negative rate, while serum CA125 has limited diagnostic accuracy. In our previous studies, circulating endometrial cells (CECs) were identified in the peripheral blood of patients with EM, suggesting their potential as a non-invasive biomarker. Building on this finding, the present study aimed to systematically evaluate the clinical value of CECs in the diagnosis and stratified diagnosis of EM in the absence of a perfect diagnostic reference standard. **Methods**: Female patients treated at the Department of Obstetrics and Gynecology, Peking University People’s Hospital, between June 2022 and June 2024 were enrolled. Participants were clinically classified according to laparoscopic evaluation into an EM group and a non-EM group. However, laparoscopy was not treated as a definitive diagnostic gold standard in the statistical analysis. Instead, given the absence of a perfect reference standard, nonparametric latent class analysis was applied to jointly estimate disease status and the diagnostic performance of CECs, CA125, and laparoscopy. Patients with EM were further stratified according to dysmenorrhea severity (mild, moderate, and severe), lesion activity status (active or dormant), and menstrual cycle phase. Peripheral blood samples were collected from all participants, and CECs were detected using subtraction enrichment combined with immunofluorescence and fluorescence in situ hybridization (SE-iFISH). Serum CA125 levels were measured concurrently. **Results**: A total of 302 participants were included. The primary analysis focused on 133 surgically confirmed EM patients and 146 non-EM controls. After adjustment for an imperfect diagnostic reference standard, CECs demonstrated superior diagnostic performance compared with serum CA125 in the overall cohort, with higher sensitivity (0.58 vs. 0.37) and specificity (0.81 vs. 0.75). Under laparoscopic assessment in patients with severe dysmenorrhea (VAS ≥ 7), where the sensitivity and specificity were 0.759 and 1.00, respectively, CECs demonstrated superior diagnostic performance compared with serum CA125, with higher sensitivity (0.694 vs. 0.355) and specificity (0.946 vs. 0.429). Similarly, in patients with active EM, where laparoscopy showed a sensitivity of 0.79 and a specificity of 1.00, CECs again demonstrated superior diagnostic performance compared with CA125 (sensitivity 0.73 vs. 0.35; specificity 0.96 vs. 0.31), showing high concordance with laparoscopic diagnosis. When stratified by menstrual cycle phase, CECs maintained superior diagnostic performance over CA125 during both the proliferative and menstrual phases, with higher sensitivity (0.84 vs. 0.44) and specificity (0.83 vs. 0.65). **Conclusions**: Circulating endometrial cells (CECs) demonstrate high diagnostic accuracy for EM, significantly outperforming serum CA125, and show high concordance with laparoscopic diagnosis across clinically relevant stratified conditions in the absence of a perfect diagnostic gold standard.

## 1. Introduction

Endometriosis (EM) is a common chronic gynecological disease affecting approximately 10–15% of women of reproductive age worldwide [1]. It is defined by the presence of endometrial-like tissue with glandular and stromal components outside the uterine cavity and is associated with chronic pelvic pain, severe dysmenorrhea, infertility, and a wide spectrum of atypical clinical manifestations [2]. The clinical presentation of EM is highly heterogeneous, and symptom severity often shows poor correlation with lesion extent [3]. Moreover, the pathogenesis of EM has not yet been fully elucidated [4], which further increases the challenges associated with its diagnosis and management [5,6,7].

Although laparoscopy has long been regarded as the diagnostic “gold standard” for EM, it has inherent limitations [8] and is highly dependent on the experience and skill of the surgeon. Subtle, atypical, subperitoneal, or extra-pelvic lesions may be missed, leading to false-negative results [9], with miss rates reaching up to 20% in certain patient populations [6]. Consequently, the 2022 ESHRE guidelines no longer consider laparoscopy the sole diagnostic standard, but instead advocate an integrated approach based on clinical presentation and imaging findings [10]. Chinese guidelines similarly emphasize the diagnostic importance of key symptoms, including pelvic pain, dysmenorrhea, and infertility [11].

In recent years, machine learning models based on symptom questionnaires and clinical characteristics have shown some effectiveness in EM risk stratification, but they remain susceptible to recall bias and lack objectivity [12,13,14,15]. Other diagnostic tools, including ultrasound, magnetic resonance imaging (MRI), and serum CA125 testing, demonstrate limited sensitivity and specificity for EM [16,17,18,19], particularly in the detection of early or minimal disease [20]. Molecular biomarkers, such as microRNAs (miRNAs), have shown promising diagnostic potential [21,22,23,24,25,26,27,28,29]; however, their clinical application remains constrained by small sample sizes and a lack of external validation. Proteomic and inflammatory markers have also been explored, with biomarkers such as MIF and cytokines including IL-6, IL-8, and IL-32 demonstrating some diagnostic value in EM [30,31,32,33,34,35,36,37,38], although their reproducibility and generalizability remain limited. Consequently, the diagnosis of EM is often delayed by 7–12 years, underscoring the urgent need for reliable and minimally invasive diagnostic approaches [39].

In our previous studies, we identified CECs in the peripheral blood of patients with EM and demonstrated their potential as a novel, non-invasive biomarker for early disease detection [40]. We further observed that higher CEC levels were associated with increased disease activity [41]. However, previous studies have been limited by small sample sizes and the lack of an ideal reference standard. As a result, the diagnostic sensitivity, specificity, and stability of CECs in real-world clinical settings have not been systematically evaluated. To address these limitations, the present study expanded the sample size and included patients with pelvic masses scheduled for surgery, with stratification based on clinically relevant subgroups. We systematically evaluated the diagnostic and stratification value of CECs in EM and compared their performance with that of laparoscopy and serum CA125. Given the lack of a perfect diagnostic gold standard, we further applied advanced statistical methods designed for imperfect reference standards to achieve a more objective assessment of CEC performance.

Overall, this study systematically evaluates the clinical value of CECs as a biomarker for the diagnosis and stratification of EM and explores methodological solutions to address the challenge of an imperfect diagnostic gold standard. CECs may help determine whether the disease is in an active or progressive state, providing valuable support for treatment selection, follow-up management, and assessment of therapeutic response, ultimately enabling more timely and rational clinical decision-making.

## 2. Materials and Methods

### 2.1. Participant Classification and Baseline Characteristics

Female patients treated at the Department of Obstetrics and Gynecology, Peking University People’s Hospital, between June 2022 and June 2024 were consecutively recruited. All participants were found to have pelvic masses during clinical evaluation and were scheduled for surgical intervention. After comprehensive screening, patients were enrolled according to the following inclusion criteria: (1) female patients with pelvic masses planned for surgical treatment and (2) voluntary participation with written informed consent. Exclusion criteria were: (1) pregnancy, lactation, or postmenopausal status and (2) diagnosed hematologic diseases or abnormal leukocyte counts.

Patients who met the clinical diagnostic criteria for EM were additionally classified into a clinically diagnosed EM group. The clinical diagnostic criteria included one or more of the following: (1) dysmenorrhea significantly affecting daily activities and quality of life; (2) chronic pelvic pain; (3) dyspareunia or postcoital pain; (4) gastrointestinal symptoms associated with the menstrual cycle, particularly dyschezia; (5) urinary tract symptoms related to the menstrual cycle, especially hematuria or dysuria; and (6) infertility accompanied by at least one of the above symptoms.

All patients underwent standardized clinical evaluation by experienced gynecologists at our institution. Menstrual cycle phase was determined by two independent clinicians using menstrual dates combined with pathological findings. The assessment was performed on the day of blood collection and surgery. Active endometriosis (active EM) was defined as dysmenorrhea that had markedly worsened over the past six months, with pain reaching a moderate-to-severe level [41]. EM staging was classified according to the revised American Society for Reproductive Medicine (rASRM) criteria.

### 2.2. Blood Sample Collection

Peripheral blood samples were collected from all participants on the morning of their scheduled surgeries, prior to any surgical procedures. A subset of patients had received preoperative gonadotropin-releasing hormone agonist (GnRHa) treatment before blood collection, and treatment status was recorded. Approximately 6 mL of venous blood was drawn into tubes containing acid citrate dextrose (ACD) anticoagulant (Becton Dickinson, Franklin Lakes, NJ, USA). Immediately after collection, the tubes were gently inverted to ensure thorough mixing of the anticoagulant with the blood. Samples were stored at room temperature in the dark and processed within 24 h to preserve cell viability.

### 2.3. Subtraction Enrichment and Identification of Circulating Endometrial Cells

Subtraction enrichment (SE) of CECs, followed by liquid-based immunofluorescence staining and fluorescence in situ hybridization (FISH), was performed using a commercially available system (Cytelligen, San Diego, CA, USA). Briefly, peripheral blood samples were processed by subtraction enrichment to remove hematopoietic cells, after which enriched cells were subjected to immunofluorescence identification and FISH-based signal analysis. Automated scanning and image acquisition were conducted using the Metafer system (MetaSystems, Altlussheim, Germany) in combination with iFISH analysis. CEC positivity was defined as the presence of at least one circulating endometrial cell per 6 mL of peripheral blood, a pre-specified threshold validated in our previous study. To minimize potential bias, all samples were anonymized and coded prior to processing, and laboratory personnel performing CEC isolation, staining, and counting were blinded to the clinical information and group assignment of the participants. Data analysis was conducted independently by a statistician using only coded datasets. Detailed procedures and methodological validation have been reported previously [41]. This method demonstrated a detection rate of 94.4% in patients with active endometriosis, compared with 16.7% in the control group, indicating good detection capability. However, due to the limited sample size, sensitivity and specificity were not systematically evaluated.

### 2.4. Statistical Analysis

All statistical analyses were conducted using R statistical software (version 4.2.0, R Foundation for Statistical Computing, Vienna, Austria) within the RStudio environment (version 2022.7.2.576, RStudio PBC, Boston, MA, USA). Nonparametric latent class analysis was applied to evaluate the diagnostic performance of surgical diagnosis, CEC detection, and serum CA125 testing. Detailed definitions of key statistical concepts and diagnostic performance measures used in this study are provided in Supplementary Table S1. To account for potential misclassification associated with surgical diagnosis, its diagnostic sensitivity was treated as an unknown parameter and estimated directly from the observed data [42,43].

The prevalence of EM and the diagnostic accuracy of the aforementioned diagnostic modalities were modeled as nonparametric functions varying with visual analogue scale (VAS) pain scores and across different menstrual cycle phases. Diagnostic sensitivity and specificity stratified by menstrual cycle phase and VAS score were calculated using the Expectation–Maximization algorithm. For analyses stratified solely by menstrual cycle phase or conducted in the overall study population, an imputation-based approach was employed to estimate diagnostic performance. All 95% confidence intervals were constructed using the bootstrap method.

## 3. Results

### 3.1. Patient Grouping Based on Postoperative Diagnosis and Clinical Characteristics

**Figure 1:**
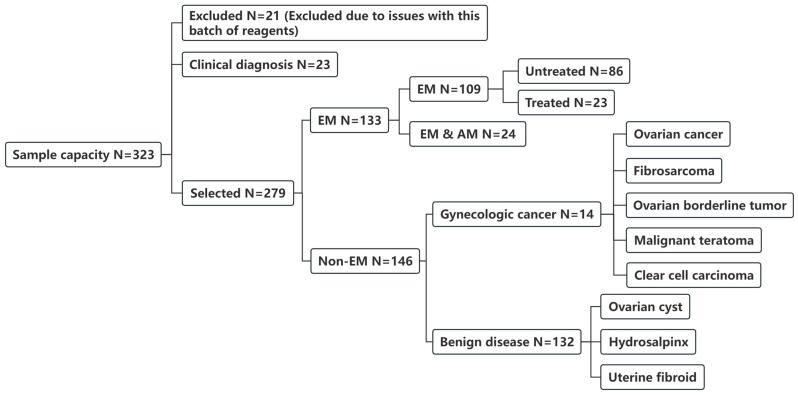
Grouping of all study subjects. The final groups included: untreated endometriosis (EM) group, endometriosis with adenomyosis (EM + AM) group, treated endometriosis group, clinically diagnosed group, benign disease group, and gynecologic malignancy group.

A total of 323 patients were initially enrolled. Due to technical issues related to antibodies used in the SE-iFISH assay, 21 patients were excluded. Ultimately, 302 patients were included in the final analysis, comprising 133 patients diagnosed with EM based on laparoscopic evaluation, 23 clinically diagnosed EM patients, and 146 non-EM control patients. Among the surgically confirmed EM patients, 86 had not received any prior treatment, 23 patients had received GnRHa treatment and 24 were diagnosed with EM concomitant with adenomyosis. The control group consisted of 146 patients, including 14 with malignant tumors and 132 with benign gynecological diseases (Figure 1).

Supplementary Table S2 summarizes the clinical and demographic characteristics of all participants, including age, menstrual cycle phase at blood collection, rASRM stage, dysmenorrhea status, VAS pain score, hemoglobin level, and relevant serum biomarkers such as CA125, CA199, and HE4.

### 3.2. Overall Detection of CECs and Diagnostic Performance in EM and Non-EM Populations

**Figure 2:**
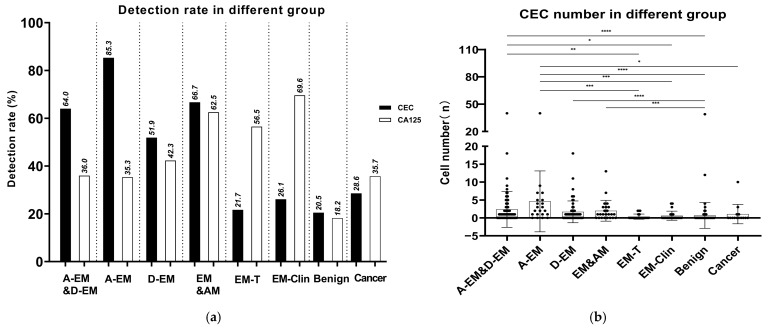
Detection rates and cell counts of circulating endometrial cells (CECs) in different patient groups. (**a**) Detection rates of CEC and CA125 positivity among different groups, including active EM (A-EM), dormant EM (D-EM), EM with adenomyosis (EM&AM), EM after medical treatment (EM-T), clinically suspected EM (EM-Clin), benign gynecological diseases (Benign), and malignant tumors (Cancer). Dotted vertical lines separate different clinical subgroups. (**b**) Average counts of CECs detected per 6 mL of peripheral blood in each patient group, expressed as mean ± standard deviation (SD). Each dot represents the number of circulating endometrial cells (CECs) detected in an individual patient. Horizontal lines indicate group comparisons, and statistical significance is denoted by asterisks (* *p* < 0.05, ** *p* < 0.01, *** *p* < 0.001, **** *p* < 0.0001).

Among the 302 subjects included in the analysis, CECs were detected at a significantly higher frequency in patients with EM than in non-EM controls (Figure 2). In surgically confirmed EM patients, regardless of whether the disease was classified as active or dormant, the overall detection rate of CECs was 64.0% (55/86), with a mean count of 2.38 cells per 6 mL of peripheral blood.

In contrast, the detection rate of CECs in the non-EM control group—including patients with benign gynecological diseases and gynecological malignancies—was 24.6% (36/146), with a mean count of 0.88 cells per 6 mL, both of which were significantly lower than those observed in EM patients.

In the overall population of 279 patients (133 EM and 146 non-EM controls) without stratification by pain severity, lesion activity, or menstrual cycle phase, diagnostic performance of CEC detection, serum CA125 testing, and laparoscopy was compared using statistical methods adjusted for an imperfect reference standard (Table 1). Laparoscopy demonstrated an overall diagnostic sensitivity of 0.88 and a specificity of 1.00. The overall diagnostic sensitivity and specificity of CEC detection were 0.58 and 0.81, respectively, both higher than those of serum CA125 (sensitivity 0.37; specificity 0.75).

### 3.3. Diagnostic Value of CECs Across Different Stratifications

#### 3.3.1. CEC Detection and Diagnostic Performance Stratified by Dysmenorrhea Severity

Stratified analysis by dysmenorrhea severity showed an increasing trend in CEC detection with higher visual analogue scale (VAS) scores. The CEC detection rate was 67.9% (19/28) in patients without dysmenorrhea (VAS = 0), 56.5% (13/23) in patients with mild dysmenorrhea (VAS 1–3), 47.4% (9/19) in patients with moderate dysmenorrhea (VAS 4–6), and increased to 86.7% (13/15) in patients with severe dysmenorrhea (VAS ≥ 7). Similarly, the mean CEC count was 1.68 and 1.48 cells per 6 mL of peripheral blood in patients without and with mild dysmenorrhea, respectively, and increased to 3.63 and 3.53 cells per 6 mL in patients with moderate and severe dysmenorrhea.

**Figure 3:**
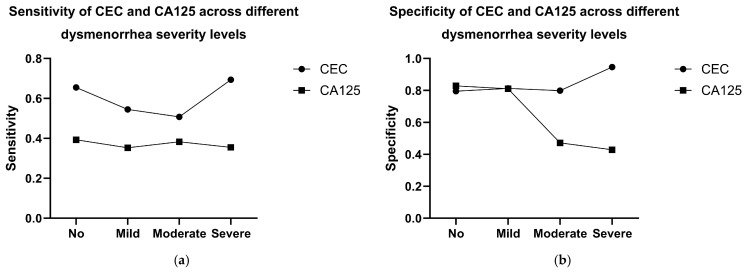
Diagnostic performance of CECs and CA125 across different dysmenorrhea severity levels in patients with endometriosis. (**a**) CECs demonstrate the highest sensitivity (0.694) in patients with endometriosis and severe dysmenorrhea (VAS ≥ 7), significantly outperforming CA125 (sensitivity 0.355); (**b**) CECs also show superior specificity (0.946) in the same subgroup compared to CA125 (specificity 0.429).

In terms of diagnostic performance, CEC detection consistently outperformed serum CA125 across all dysmenorrhea severity strata. In patients without dysmenorrhea (VAS = 0), CECs showed a sensitivity of 0.655 and a specificity of 0.795, compared with 0.393 and 0.829 for CA125. Similar superiority was observed in patients with mild dysmenorrhea (VAS 1–3), with CEC sensitivity and specificity of 0.545 and 0.813, respectively, versus 0.353 and 0.810 for CA125. In patients with moderate dysmenorrhea (VAS 4–6), CEC detection achieved a sensitivity of 0.508 and a specificity of 0.799, exceeding the performance of CA125 (0.383 and 0.472). The highest diagnostic performance was observed in patients with severe dysmenorrhea (VAS ≥ 7), where CECs demonstrated a sensitivity of 0.694 and a specificity of 0.946, markedly superior to CA125 (0.355 and 0.429) and comparable to laparoscopic diagnosis (0.759 and 1.000) (Figure 3 and Table 2).

#### 3.3.2. CEC Detection and Diagnostic Performance Stratified by Lesion Activity

Stratified analysis based on lesion activity demonstrated significant differences in CEC detection across distinct disease states. Compared with dormant EM patients and non-EM controls, patients with active EM exhibited a markedly higher CEC detection rate, with rates of 85.3% (15/18), 51.9% (35/67), and 24.0% (35/146), respectively. Correspondingly, the mean CEC count was significantly increased in active EM patients compared with dormant EM patients and non-EM controls (4.62 vs. 1.69 vs. 0.88 cells/6 mL, respectively; Figure 2).

Within the statistical framework adjusted for an imperfect diagnostic reference standard, diagnostic performance of CEC detection, serum CA125 testing, and laparoscopy was further compared. In patients with active EM, CEC detection achieved a diagnostic sensitivity of 0.734 and a specificity of 0.961, which were substantially higher than those of serum CA125 (sensitivity 0.352, specificity 0.311). Laparoscopy demonstrated a sensitivity of 0.791 and a specificity of 1.000 in this subgroup, showing high concordance with CEC-based diagnosis (Table 3).

In patients with dormant EM, both the sensitivity and specificity of CEC detection were reduced compared with those observed in active EM patients; however, overall diagnostic performance of CECs remained superior to that of serum CA125. Similarly, diagnostic performance of laparoscopy was also reduced in this subgroup (Table 3).

#### 3.3.3. CEC Detection and Diagnostic Performance Across Different Menstrual Cycle Phases

**Figure 4:**
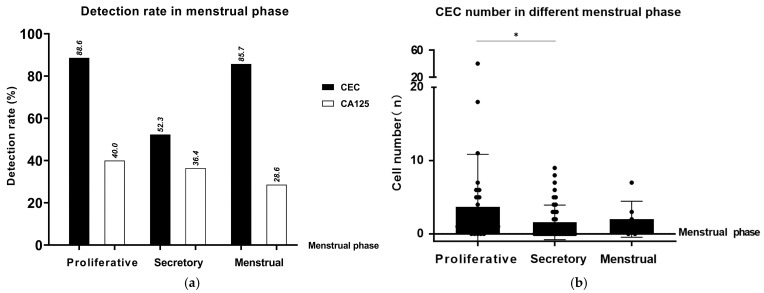
CEC detection across different phases of the menstrual cycle. (**a**) Comparison of CEC detection rates and CA125 positivity across menstrual phases. CECs showed the highest detection rate during the proliferative phase (88.6%), compared to lower CA125 positivity (40%). During the secretory phase, CEC detection was 52.3% and CA125 was 36.4%. In the menstrual phase, CEC detection remained high at 85.7%, while CA125 positivity dropped to 28.6%. (**b**) Quantitative analysis of CEC across menstrual phases. Each dot represents the number of circulating endometrial cells (CECs) detected in an individual patient. The average CEC count was highest during the proliferative phase (3.69 cells/6 mL), followed by the menstrual phase (3.11 cells/6 mL), and lowest in the secretory phase (1.58 cells/6 mL), indicating cyclical variation in CEC levels. Horizontal lines indicate group comparisons, and statistical significance is denoted by asterisks (* *p* < 0.05).

Stratified analysis by menstrual cycle phase revealed significant variations in both CEC detection and diagnostic performance. The detection rates of CECs during the proliferative phase and menstrual phase were 88.6% and 85.7%, respectively, which were significantly higher than that observed during the secretory phase (52.3%). Likewise, mean CEC counts were markedly increased in the proliferative and menstrual phases compared with the secretory phase (3.69 and 3.11 vs. 1.58 cells/6 mL, respectively; Figure 4).

Analysis of diagnostic sensitivity and specificity demonstrated that during the proliferative and menstrual phases, CEC detection achieved a sensitivity of 0.84 and a specificity of 0.83, both of which were higher than those observed during the secretory phase (sensitivity 0.52, specificity 0.82) and superior to those of concurrent serum CA125 testing (Table 4). Laparoscopy maintained consistently high specificity (1.00) across all menstrual cycle phases; however, its sensitivity declined from 0.99 in the proliferative and menstrual phase to 0.91 in the secretory phase.

**Figure 5:**
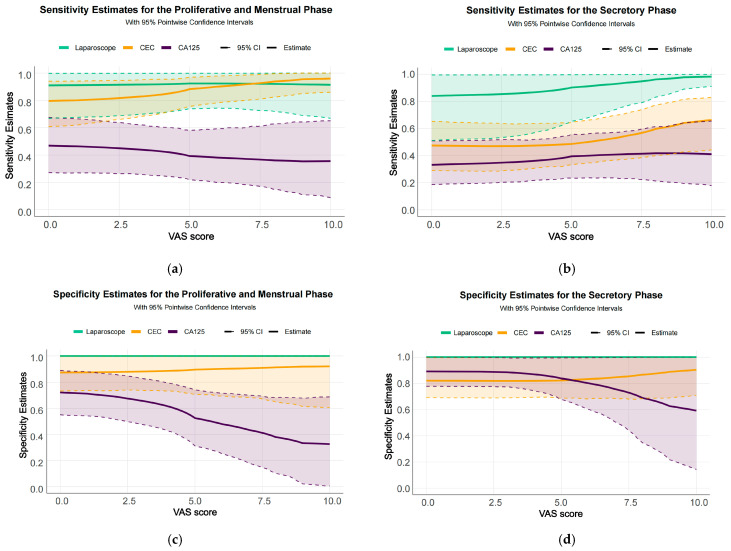
Diagnostic Ability Curves of CECs, CA125, and Laparoscopy Across Different Menstrual Phases and VAS Pain Score Groups. Solid lines represent estimates, and dashed lines indicate 95% confidence intervals (95% CI). (**a**) Sensitivity curve in the proliferative and menstrual phase, showing that CECs perform comparably to laparoscopy and significantly outperform CA125, especially in patients with VAS scores above 7.5. (**b**) Sensitivity curve in the secretory phase, where CECs remain superior to CA125, though with slightly reduced sensitivity compared to the proliferative phase. (**c**) Specificity curve in the proliferative and menstrual phase, demonstrating that CECs consistently outperform CA125, with minimal impact from VAS scores, maintaining high specificity even in patients with severe dysmenorrhea. (**d**) Specificity curve in the secretory phase, with CECs showing stable specificity, maintaining high specificity even in the group with severe pain.

To further characterize diagnostic performance across menstrual cycle phases and pain severity strata, comprehensive statistical analyses were performed, and sensitivity and specificity curves were generated for CECs, serum CA125, and laparoscopy. During the proliferative and menstrual phase (Figure 5a), the diagnostic sensitivity of CECs was comparable to that of laparoscopy and significantly higher than that of CA125. Notably, when VAS pain scores exceeded 7.5, CECs showed a slightly higher sensitivity than laparoscopy; however, this difference did not reach statistical significance. In contrast, during the secretory phase (Figure 5b), although the sensitivity of CEC detection was modestly reduced compared with the proliferative phase, it remained markedly superior to CA125, indicating that CECs retain robust diagnostic capability across different menstrual cycle phases.

Further evaluation of specificity showed that during the proliferative and menstrual phase (Figure 5c), CEC detection consistently exhibited higher specificity than CA125, with minimal variation across different VAS pain score ranges. Similarly, during the secretory phase (Figure 5d), CEC specificity remained relatively stable and high, even in patients with severe pain.

#### 3.3.4. CEC Detection Characteristics in EM Patients During Medical Treatment

Among the 23 EM patients receiving medical treatment at the time of CEC assessment, marked differences in CEC detection were observed compared with untreated EM patients. The detection rate of CECs in treated patients was 21.7% (5/23), which was significantly lower than that observed in untreated EM patients (64.0%, 55/86; Figure 2). In contrast, serum CA125 remained positive in a substantial proportion of treated patients (56.5%, 13/23), exhibiting a detection pattern distinct from that of CECs. These findings indicate that CEC detection is closely associated with treatment status and appears to be more sensitive to therapeutic effects than CA125, suggesting a potential role for CECs in monitoring treatment response in EM.

#### 3.3.5. CEC Subtype Characteristics Among Different Patient Groups

**Figure 6:**
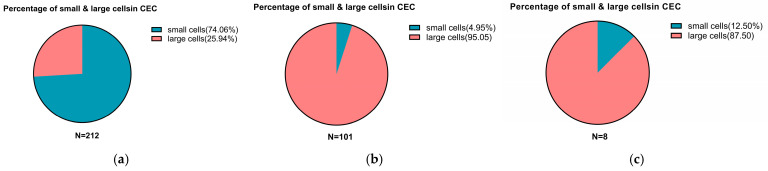
Subtype characterization of circulating endometrial cells (CECs) among EM, non-EM controls, and EM patients after medical treatment. Detailed subtype analysis was performed based on cell size, with cells classified as small (<5 μm) or large (≥5 μm). (**a**) EM group: CECs were predominantly small-sized cells (74.06%). (**b**) Non-EM control group: CECs were predominantly large-sized cells (95.05%); (**c**) EM post-treatment group: Predominantly large-sized cells (87.50%).

Analysis of CEC subtypes revealed significant differences between EM patients and control populations. In surgically confirmed EM patients, small-sized CECs (diameter < 5 μm) predominated, accounting for 74.06% of detected cells. In contrast, large-sized CECs (≥5 μm) were predominant in non-EM controls and in EM patients following treatment, representing 95.05% and 87.50% of detected cells, respectively (Figure 6). These findings demonstrate distinct differences in CEC size distribution among patient groups, suggesting biological heterogeneity within CEC subpopulations and highlighting their potential clinical utility in the diagnosis and monitoring of EM.

## 4. Discussion

EM is a heterogeneous disorder, with poor correlation between lesion extent and symptom severity, as limited lesions may cause severe symptoms while extensive disease may be mildly symptomatic [44]. This dissociation complicates clinical assessment. It also supports stratification based on pain severity, disease activity, and menstrual cycle phase to guide individualized management, prioritizing intervention for active disease and monitoring for quiescent cases [45]. However, such stratification strategies have rarely been systematically explored in previous studies.

One of the central challenges in EM diagnosis lies in the absence of a universally accepted diagnostic “gold standard.” Although laparoscopy is widely regarded as the reference standard for EM, its limited sensitivity for small or deeply infiltrating lesions may result in false negatives [46]. Consequently, recent clinical guidelines no longer consider laparoscopy an absolute diagnostic standard. In this context, the present study evaluated the diagnostic and stratified value of CECs using statistical approaches designed for imperfect reference standards. Notably, the relatively wide confidence intervals observed in some analyses likely reflect model complexity rather than imprecision. This model accounts for the imperfect reference standard while incorporating covariates such as VAS scores using nonparametric methods [47]. Statistical theory indicates that models with more parameters or nonparametric methods tend to produce wider confidence intervals, reflecting model complexity rather than reduced reliability. This approach allows an objective evaluation of CEC diagnostic sensitivity and specificity across clinically relevant dimensions, including pain severity, lesion activity, and menstrual cycle phase.

Our results demonstrated that the diagnostic performance of CECs was superior to that of serum CA125 and, in certain stratified settings, showed high concordance with laparoscopic diagnosis. Serum CA125, although widely used in clinical practice, has long been limited by insufficient sensitivity and specificity, particularly in early-stage or mild EM, thereby restricting its diagnostic utility.

Dysmenorrhea is one of the most common and clinically meaningful symptoms of EM; however, its severity does not always correlate linearly with lesion burden or disease activity. In the present study, dysmenorrhea severity appeared to influence the diagnostic performance of different modalities. Notably, laparoscopy demonstrated high sensitivity (0.956) in patients with moderate dysmenorrhea, supporting its diagnostic value in this clinical context. Meanwhile, the diagnostic performance of CECs varied according to dysmenorrhea severity and reached its highest level in patients with severe dysmenorrhea (visual analogue scale, VAS ≥ 7), with a sensitivity of 0.694 and a specificity of 0.946. These findings indicate that CEC levels are closely associated with disease-related biological activity rather than merely reflecting subjective pain perception. Therefore, CEC detection may serve as an objective tool for distinguishing EM patients with different degrees of dysmenorrhea severity.

Further stratification based on lesion activity status revealed that both the detection rate and number of CECs were significantly higher in patients with active EM compared with those with dormant EM and non-EM controls. These findings are consistent with our previous study. Biologically, active EM is often accompanied by recent symptom exacerbation, enhanced local inflammatory responses, and instability of the lesion microenvironment. Such conditions may facilitate shedding, invasion, or hematogenous dissemination of ectopic endometrial tissue into the peripheral circulation, resulting in increased CEC levels. Therefore, CEC elevation may reflect not only the presence of endometriotic lesions but also their ongoing biological activity. This characteristic suggests that CECs may serve as a biomarker of disease activity and biological behavior, in addition to their diagnostic utility. It is important to note that the criteria used to define lesion activity in this study differ from those used in our previous work. In our earlier study, active EM was defined by symptom exacerbation over the preceding six months combined with imaging evidence of significant ovarian cyst enlargement (>2 cm). However, evidence suggests that some EM patients may harbor small or occult lesions that are difficult to detect by laparoscopy or imaging, while biological progression and symptom worsening continue. In such cases, reliance on visible lesion size alone may fail to identify a subset of patients with ongoing disease progression. Meanwhile, updated guidelines no longer consider laparoscopy the sole diagnostic gold standard and stress symptom-based assessment. In line with this paradigm shift, active EM in this study was defined by significant worsening of dysmenorrhea over the past six months and moderate-to-severe pain (VAS ≥ 6), enabling more sensitive identification of patients without relying on visible lesion changes. Despite differences in criteria between studies, the objective remains to comprehensively identify EM patients with ongoing disease progression.

The influence of the menstrual cycle on EM-related biomarkers has long attracted considerable attention. In the present study, the detection rate and absolute number of circulating endometrial cells (CECs) were significantly higher during the proliferative and menstrual phases than during the secretory phase. Due to the limited number of participants sampled during the menstrual phase, independent analysis of this phase was not feasible. Given comparable CEC levels between the menstrual and proliferative phases, these phases were pooled to enhance statistical robustness, although reliability may still be affected by sample size. The results indicate that CEC diagnostic performance varies across the menstrual cycle, with higher sensitivity observed in the proliferative and menstrual phases and lower sensitivity in the secretory phase, while specificity remained relatively stable. Within each phase, CEC performance was consistent across different levels of dysmenorrhea, suggesting minimal influence from subjective symptoms. By contrast, serum CA125 showed overall lower diagnostic performance and was more susceptible to menstrual cycle and pain severity, indicating that CECs may more stably reflect disease-related biological processes. Notably, although CEC levels may fluctuate across the menstrual cycle, no cycle standardization or further stratification was applied due to the limited sample size in order to avoid excessively small subgroups and reduced statistical power. Overall, these findings indicate that the menstrual cycle exerts a substantial influence on the dynamic characteristics of CECs and suggest that the timing of blood sample collection within the menstrual cycle may be an important consideration in clinical practice.

In EM patients receiving medical treatment, both the detection rate and the number of CECs were markedly reduced, whereas serum CA125 remained positive at relatively high rates during treatment. Although the number of treated patients included in this study was limited and the analysis was primarily cross-sectional, these findings suggest that CECs may be more sensitive to treatment-related changes. The post-treatment decline in CEC levels may reflect attenuation of lesion biological activity, indicating their potential value in disease monitoring and treatment response assessment. This observation provides an important rationale for future longitudinal studies using CECs as indicators of therapeutic response.

Subtype analysis of CECs further revealed substantial differences between EM patients and control populations in terms of cell size. Small-sized CECs predominated in EM patients, whereas large-sized CECs were mainly observed in non-EM controls and in EM patients after treatment. From a biological perspective, smaller CECs may have a greater capacity to enter the circulation and disseminate systemically [48,49]. Compared with larger cells, smaller cells could potentially traverse microvascular barriers, adapt to the circulatory microenvironment, and partially evade immune clearance, thereby increasing their detectability in peripheral blood. This feature was particularly evident in patients with active EM. Conversely, the relative enrichment of large-sized CECs in non-EM controls and treated patients may reflect reduced dissemination potential or more efficient clearance from the circulation. It should be noted that this interpretation is speculative and requires further investigation for validation.

Nevertheless, future multicenter studies involving a larger overall sample size and more extensive subgroup populations are warranted to allow more rigorous stratified analyses. In particular, increasing the number of patients in the menstrual-phase subgroup will enable a more precise assessment of CEC diagnostic performance across different phases of the menstrual cycle. Furthermore, longitudinal follow-up studies are required to further clarify the role of CECs in disease monitoring and treatment response assessment.

## 5. Conclusions

This study systematically demonstrates the clinical value of circulating endometrial cells (CECs) as a non-invasive biomarker for the diagnosis and stratified assessment of EM. The diagnostic performance of CEC detection is superior to that of serum CA125 and shows high concordance with laparoscopic findings, particularly in patients with severe dysmenorrhea and active disease and during specific phases of the menstrual cycle.

## Figures and Tables

**Table 1:** Diagnostic accuracy of laparoscopy, CEC, and CA125 in patients with EM (non-stratified analysis). CEC: circulating endometrial cell, CA125: cancer antigen 125.

The Overall Population (*n* = 279)					
Laparoscope		CEC		CA125	
Sensitivity	Specificity	Sensitivity	Specificity	Sensitivity	Specificity
0.88	1.00	0.58	0.81	0.37	0.754
(0.69, 1.00)	(1.00, 1.00)	(0.50, 0.72)	(0.74, 0.97)	(0.26, 0.48)	(0.66, 0.86)

**Table 2:** Diagnostic accuracy of CEC detection, CA125, and laparoscopy in endometriosis patients stratified by dysmenorrhea severity. CEC: circulating endometrial cell, CA125: cancer antigen 125.

No Dysmenorrhea (VAS = 0, *n* = 107)					
Laparoscope		CEC		CA125	
Sensitivity	Specificity	Sensitivity	Specificity	Sensitivity	Specificity
0.795	1.000	0.655	0.795	0.393	0.829
(0.500, 0.989)	(1.000, 1.000)	(0.501, 0.843)	(0.678, 0.955)	(0.239, 0.582)	(0.722, 0.951)
Mild Dysmenorrhea (VAS: 1–3, *n* = 62)					
Laparoscope		CEC		CA125	
Sensitivity	Specificity	Sensitivity	Specificity	Sensitivity	Specificity
0.819	1.000	0.545	0.813	0.353	0.810
(0.575, 0.991)	(1.000, 1.000)	(0.411, 0.717)	(0.714, 0.966)	(0.227, 0.518)	(0.712, 0.929)
Moderate Dysmenorrhea (VAS: 4–6, *n* = 31)					
Laparoscope		CEC		CA125	
Sensitivity	Specificity	Sensitivity	Specificity	Sensitivity	Specificity
0.956	1.000	0.508	0.799	0.383	0.472
(0.815, 0.999)	(1.000, 1.000)	(0.290, 0.726)	(0.448, 1.000)	(0.168, 0.619)	(0.007, 0.999)
Severe Dysmenorrhea (VAS: 7–10, *n* = 29)					
Laparoscope		CEC		CA125	
Sensitivity	Specificity	Sensitivity	Specificity	Sensitivity	Specificity
0.759	1.000	0.694	0.946	0.355	0.429
(0.655, 0.999)	(1.000, 1.000)	(0.551, 0.998)	(0.706, 1.000)	(0.135, 0.611)	(0.002, 0.874)

**Table 3:** Diagnostic accuracy (sensitivity and specificity) of laparoscopy, CECs, and CA125 in patients with endometriosis (active EM vs. dormant EM). EM: endometriosis, CEC: circulating endometrial cell, CA125: cancer antigen 125.

Active Endometriosis (*n* = 35)					
Laparoscope		CEC		CA125	
Sensitivity	Specificity	Sensitivity	Specificity	Sensitivity	Specificity
0.79	1.00	0.73	0.96	0.35	0.31
(0.70, 1.00)	(1.00, 1.00)	(0.63, 1.00)	(0.76, 1.00)	(0.16, 0.58)	(0.00, 0.76)
		Dormant Endometriosis (*n* = 194)			
Laparoscope		CEC		CA125	
Sensitivity	Specificity	Sensitivity	Specificity	Sensitivity	Specificity
0.86	1.00	0.54	0.82	0.37	0.80
(0.66, 0.99)	(1.00, 1.00)	(0.44, 0.68)	(0.73, 0.97)	(0.25, 0.50)	(0.71, 0.90)

**Table 4:** Diagnostic accuracy (sensitivity and specificity) of laparoscopy CECs and CA125 in endometriosis patients stratified by menstrual cycle phases (proliferative and menstrual vs. secretory phases). CEC: circulating endometrial cell, CA125: cancer antigen 125.

The Proliferative and Menstrual Phase (*n* = 98)					
Laparoscope		CEC		CA125	
Sensitivity	Specificity	Sensitivity	Specificity	Sensitivity	Specificity
0.99	1.00	0.84	0.83	0.44	0.65
(0.01, 1.00)	(1.00, 1.00)	(0.72, 0.97)	(0.29, 1.00)	(0.25, 0.62)	(0.50, 0.80)
	The Secretory Phase (*n* = 110)				
Laparoscope		CEC		CA125	
Sensitivity	Specificity	Sensitivity	Specificity	Sensitivity	Specificity
0.91	1.00	0.52	0.82	0.36	0.87
(0.39, 1.00)	(1.00, 1.00)	(0.38, 0.67)	(0.60, 1.00)	(0.22, 0.51)	(0.71, 1.00)

## Data Availability

The data supporting the findings of this study are available from the corresponding author upon reasonable request.

## Supplementary Materials

The following supporting information can be downloaded at: https://www.mdpi.com/article/10.3390/jcm15083021/s1, Table S1: Terminology and definitions of key statistical concepts and diagnostic performance measures. Table S2: Clinical characteristics of the study participants stratified by postoperative diagnosis.

## Abbreviations

The following abbreviations are used in this manuscript:

EM	Endometriosis
CEC	Circulating Endometrial Cell
VAS	Visual Analogue Scale
