# Supplementary material for: Clinical Value of Circulating Endometrial Cells in the Diagnosis and Stratified Diagnosis of Endometriosis
Source: J Clin Med. 2026 Apr 15;15(8):3021. doi: 10.3390/jcm15083021 (PMC13115985; doi:10.3390/jcm15083021)
Supplement: Supplementary file 1 [file jcm-15-03021-s001.zip › jcm-4188890-supplementary.pdf]

Supplementary Table S1. Terminology and definitions of key statistical concepts and diagnostic performance measures.

Term	Definition
Endometriosis (EM)	An estrogen-dependent gynecological disorder characterized by the presence of endometrial-like tissue with glandular and stromal components outside the uterine cavity, accompanied by chronic inflammatory responses.
Adenomyosis (AM)	A benign gynecological condition defined by the invasion of endometrial glands and stroma into the myometrium, accompanied by reactive myometrial hyperplasia and hypertrophy.
Active Endometriosis (A-EM)	Endometriosis characterized by a marked aggravation of dysmenorrhea within the past 6 months, with a visual analogue scale (VAS) score ≥ 6.
Dormant Endometriosis (D-EM)	Endometriosis with no significant aggravation of dysmenorrhea within the past 6 months and a VAS score < 6.
Treated Endometriosis (EM-T)	Patients with endometriosis who have received medical treatment within the past 6 months.
Clinically Diagnosed Endometriosis (EM-clin)	Patients fulfilling the diagnostic criteria of the Endometriosis Diagnosis and Treatment Guidelines (3rd edition) but without surgical confirmation.
Endometriosis with Adenomyosis (EM & AM)	Patients with surgically confirmed endometriotic lesions concomitant with adenomyosis.
Imperfect Gold Standard	A reference diagnostic method used to determine the true disease status that is not 100% accurate and carries a non-negligible risk of false-negative and/or false-positive results. In this study, laparoscopy had a specificity of 100% but a sensitivity less than 100%, indicating the presence of false-negative cases.
Detection Rate	The proportion of individuals with positive test results among a specific population undergoing the test.
Sensitivity	The proportion of true-positive results among all individuals who truly have the disease.
Specificity	The proportion of true-negative results among all individuals who truly do not have the disease.
False Positive	An individual who does not truly have the disease but is incorrectly classified as positive by the diagnostic test.
False Negative	An individual who truly has the disease but is incorrectly classified as negative by the diagnostic test.

Supplementary Table S2. Clinical characteristics of the study participants stratified by postoperative diagnosis. Detailed clinical information, including menstrual cycle phase, dysmenorrhea (VAS scores), serum CA125 levels, and other relevant clinical parameters were recorded from a total of 302 evaluable participants (133 patients with surgically confirmed EM, 146 non-EM controls, and 23 clinically diagnosed but not surgically confirmed EM patients).

		Controls（n=146）		Cases（n=156）			
				Surgical diagnosis（n=133）			
		Gynecologic Cancer (n=14)	Benign disease (n=132)	untreated (n=86)	Treat (n=23)	Combined with Adenomyosis (n=24)	Clinical diagnosis (n=23)
Age years (mean ± SD)		36.93（5.54）	33.57（8.14）	34.44（6.89）	36.78（6.17）	36.7（6.16）	31.65（3.83）
Menstrual staging	proliferative stage	7（50%）	41（37.61%）	37（43.02%）	4（23.53%）	14（58.33%）	4（21.05%）
	secretory phase	6（42.9%）	59（54.13%）	44（51.16%）	11 (64.71%)	8（33.33%）	13（68.42%）
	menstrual period	1（7.1%）	9（8.26%）	5（5.82%）	2（11.76%）	2（8.34%）	2（10.53%）
dysmenorrhea		2（15.38%）	63（48.46%）	58（67.44%）	14 (60.87%)	20（83.33%）	22（95.65%）
VAS (score)		0.36 (0.93, 0-3)	1.99 (2.88, 0-10)	2.69（2.64，0-10)	2.30（2.42，0-8)	3.13 (2.92，0-8)	6.43 (2.66, 0-10)
G (n)		1.43 (1.02, 0-3)	1.06 (1.28, 0-7)	1.03（1.30，0-7)	1.05（1.00，0-3)	1.02 (1.21，0-5)	0.90 (1.26, 0-4)
P (n)		1.07 (0.83, 0-3)	0.62 (0.77, 0-3)	0.64（0.76，0-4)	0.55（0.51，0-1)	0.57 (0.52，0-4)	0.57 (0.75, 0-2)
CA125 (U/ml)		202.64（296.84，12-740)	36.29 (65.05, 4.93-478)	70.64 (113.08, 8.40-991)	106.75（174.18，13.8-753)	84.38（131.12，13.5-343)	66.74 (80.68, 18.3-406)
Ultrasound diagnosis		0	3（3.30%）	30（31.91%）	7（35%）	10（41.67%）	3（37.5%）

Note: G, gestation; P, parturition.
